# Supplementary figures and images for: Heat shock protein 90 is involved in the regulation of HMGA2-driven growth and epithelial-to-mesenchymal transition of colorectal cancer cells
Source: PeerJ. 2016 Feb 11;4:e1683. doi: 10.7717/peerj.1683 (PMC4756735; doi:10.7717/peerj.1683)

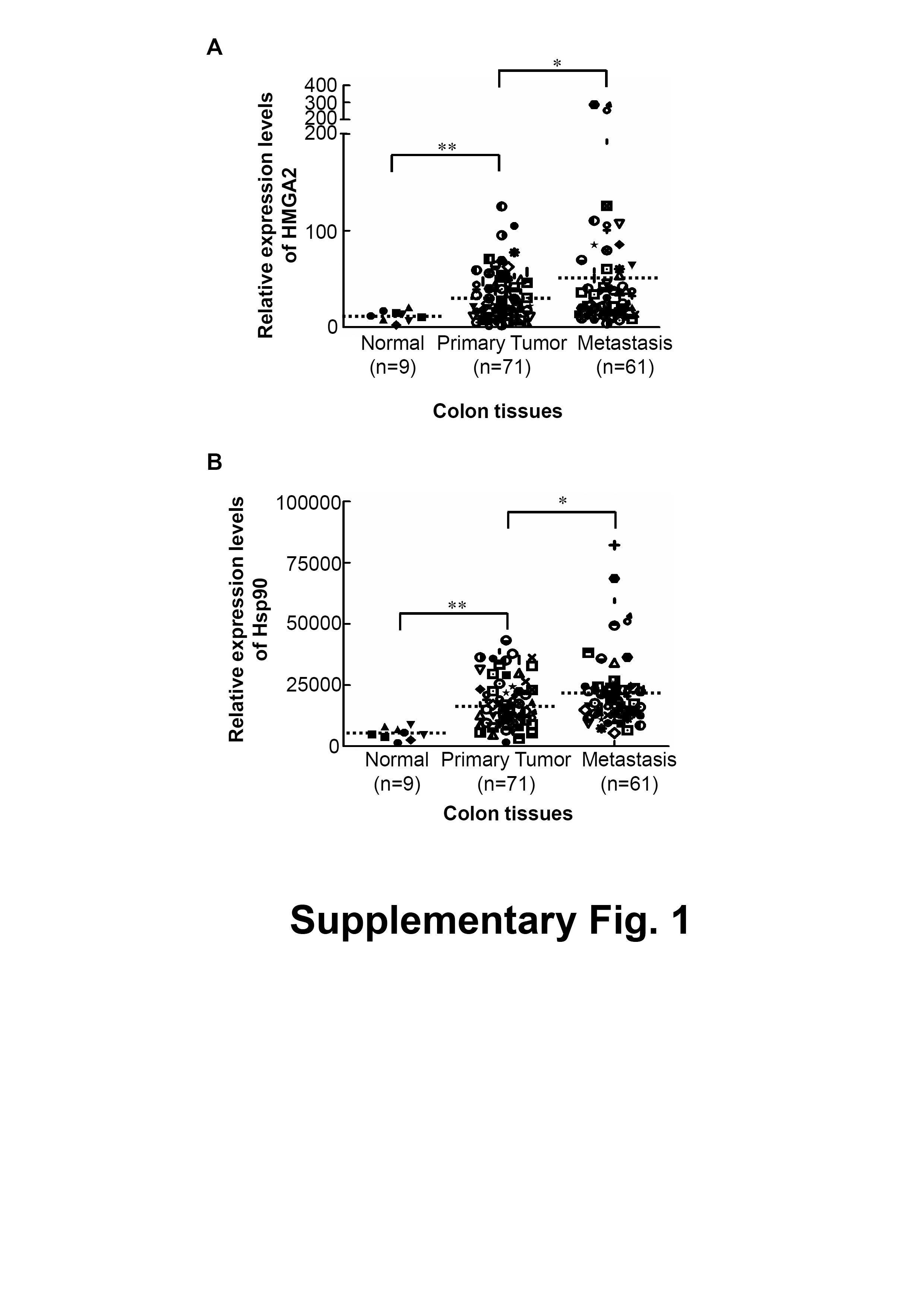

Supplement: Supplemental Information 2 — Relative expression levels of HMGA2 (A) and Hsp90 (B) at different clinical stages of CRC tissues analyzed using the public Gene Expression Omnibus database. *p < 0.05, **p < 0.01, ***p < 0.001. [file peerj-04-1683-s002.png]

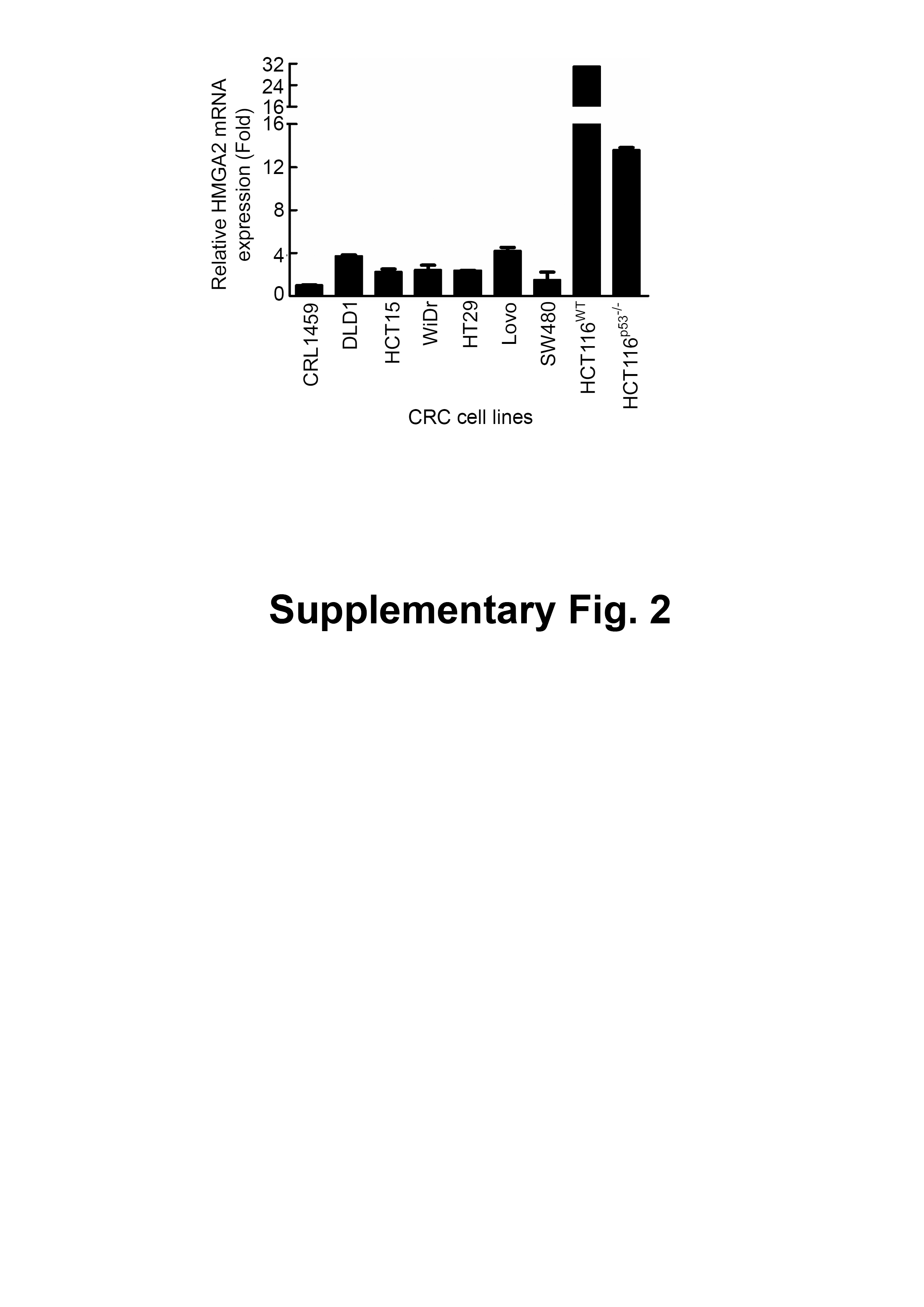

Supplement: Supplemental Information 3 [file peerj-04-1683-s003.png]

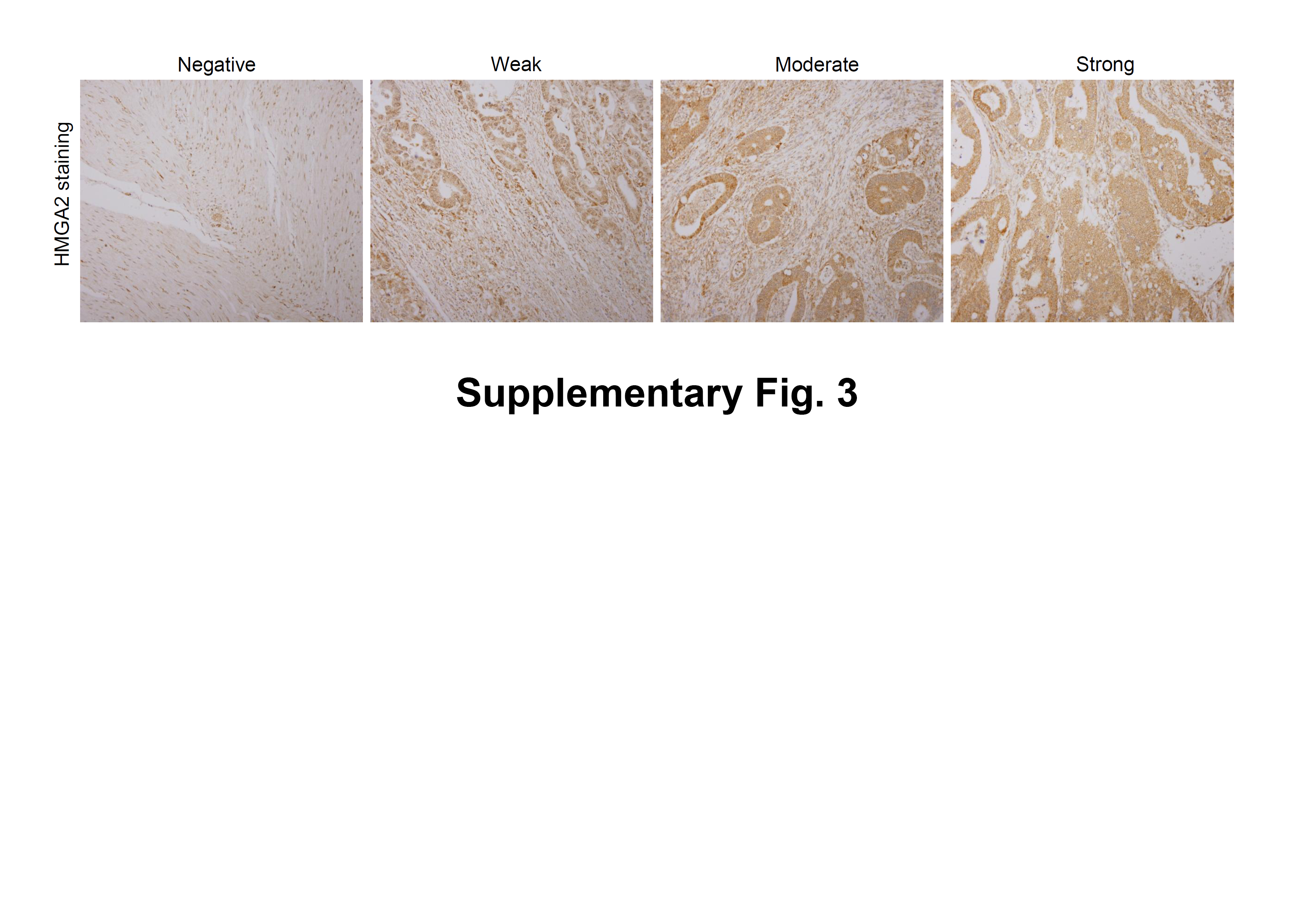

Supplement: Supplemental Information 4 [file peerj-04-1683-s004.png]

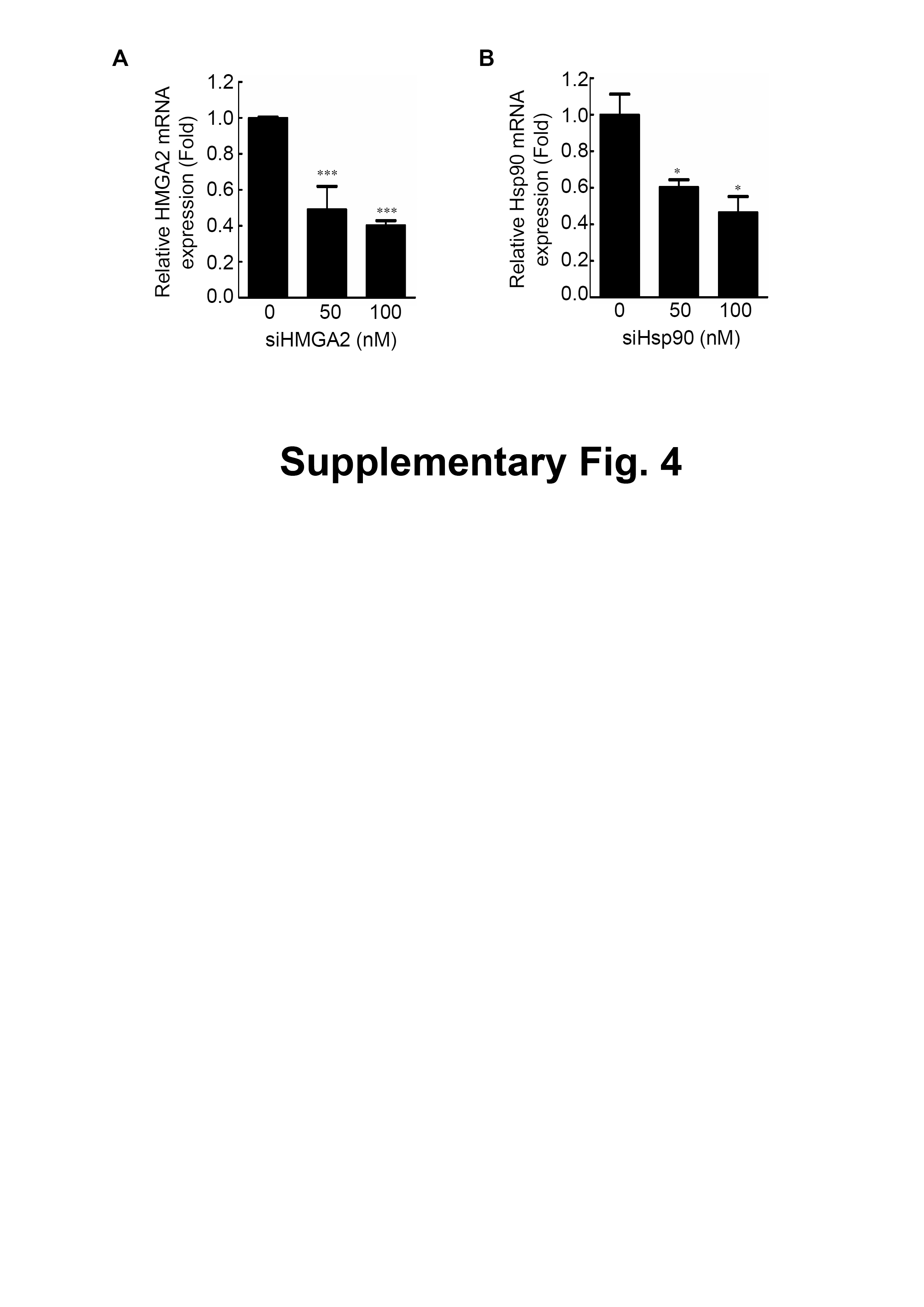

Supplement: Supplemental Information 5 [file peerj-04-1683-s005.png]

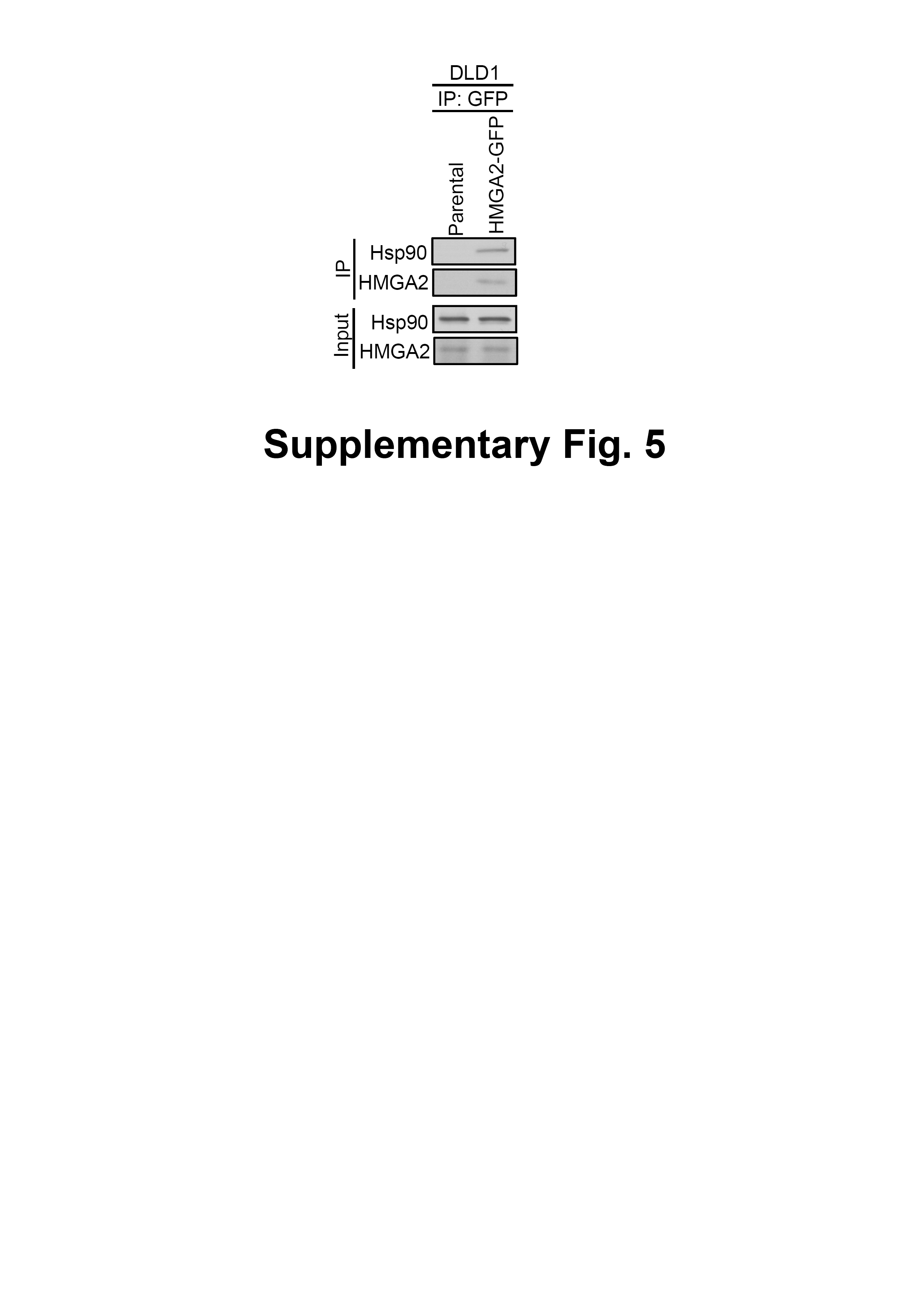

Supplement: Supplemental Information 6 — Equal amounts protein extracts of DLD1 parental cells or DLD1 HMGA2-GFP cells were Immunoprecipitated (IP) with anti-GFP antibody, and immunoprecipitates were subjected to SDS-PAGE and Immunoblotted (IB) for HMGA2 and Hsp90. Input represents of the total protein extract used for immunoprecipitation. [file peerj-04-1683-s006.png]

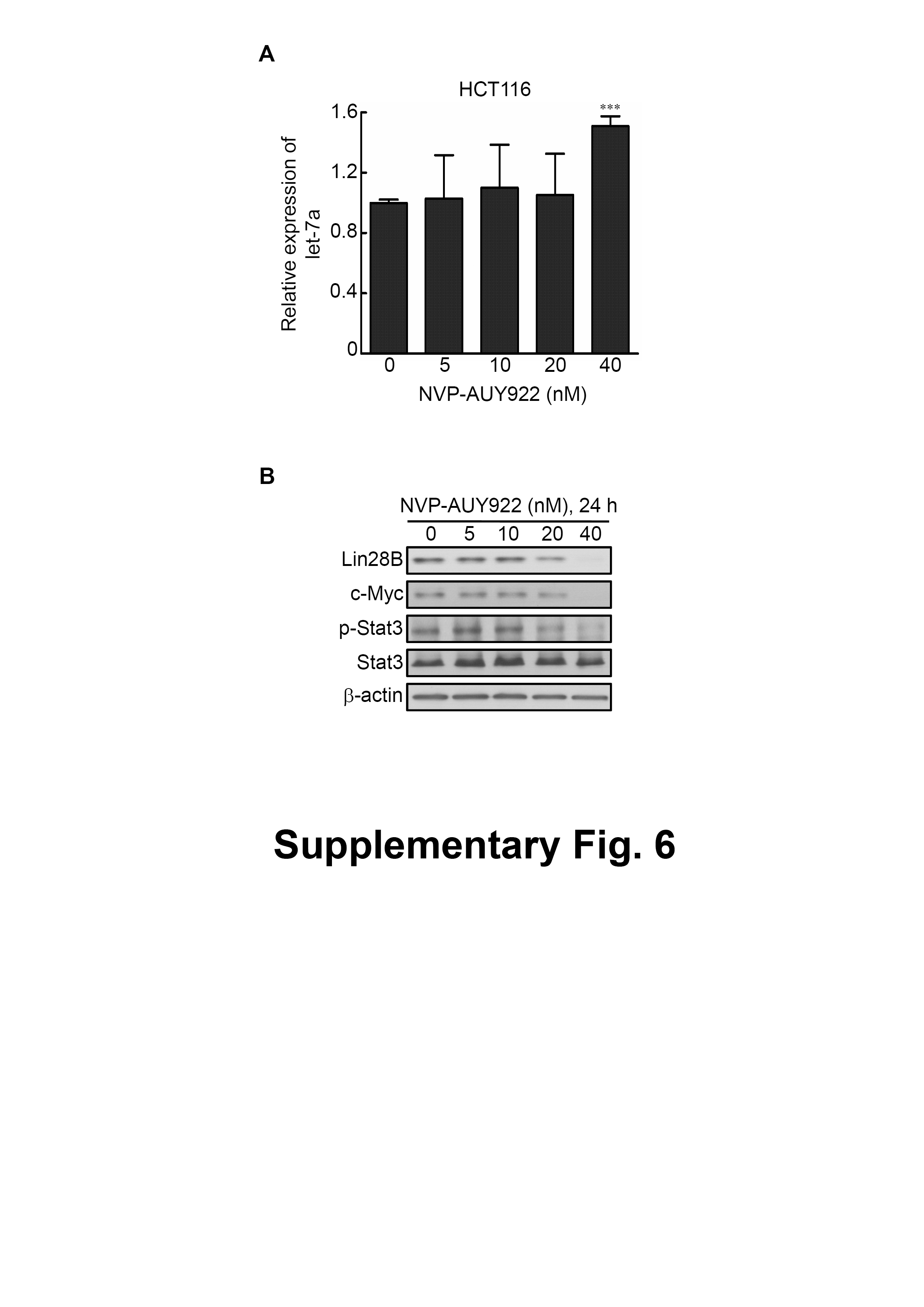

Supplement: Supplemental Information 7 — (A) Let-7a expression in HCT116 cells treated with NVP-AUY922 at the indicated concentrations for 24 h was analyzed using quantitative RT-PCR. Let-7a expression was significantly upregulated on NVP-AUY922 treatment for 40 nM. *** p < 0.001. (B) The phosphorylation of Stat3 and protein expression of Lin28B and c-myc were completely inhibited on NVP-AUY922-treated HCT116 cells in 40 nM for 24 hours. [file peerj-04-1683-s007.png]
